# Coupling SAR and optical remote sensing data for soil moisture retrieval over dense vegetation covered areas

**DOI:** 10.1371/journal.pone.0315971

**Published:** 2025-01-16

**Authors:** Jiahao Shi, Huan Yang, Xinli Hou, Honglu Zhang, Guozhong Tang, Heng Zhao, Fuqiang Wang

**Affiliations:** 1 North China University of Water Resources and Electric Power, Zhengzhou, China; 2 Key Laboratory of Conservation and Intensive Use of Water Resources in the Yellow River Basin of Henan Province, Zhengzhou, China; Sejong University, REPUBLIC OF KOREA

## Abstract

Soil moisture is a key parameter for the exchange of substance and energy at the land-air interface, timely and accurate acquisition of soil moisture is of great significance for drought monitoring, water resource management, and crop yield estimation. Synthetic aperture radar (SAR) is sensitive to soil moisture, but the effects of vegetation on SAR signals poses challenges for soil moisture retrieval in areas covered with vegetation. In this study, based on Sentinel-1 SAR and Sentinel-2 optical remote sensing data, a coupling approach was employed to retrieval surface soil moisture over dense vegetated areas. Different vegetation indices were extracted from Sentinel-2 data to establish the vegetation water content (VWC) estimation model, which was integrated with the Water Cloud Model (WCM) to distinguish the contribution of vegetation layer and soil layer to SAR backscattering signals. Subsequently, the Oh model and the Look-Up Table (LUT) algorithm were used for soil moisture retrieval, and the accuracy of the result was compared with the traditional direct retrieval method. The results indicate that, for densely vegetated surfaces, VWC can be better reflected by multiple vegetation indices including NDVI, NDWI2, NDGI and FVI, the R^2^ and RMSE of VWC estimation result is 0.709 and 0.30 kg·m^-2^. After vegetation correction, the correlation coefficient increased from 0.659 to 0.802 for the VV polarization, and from 0.398 to 0.509 for the VH polarization. Satisfactory accuracy of soil moisture retrieval result was obtained with the Oh model and the LUT algorithm, VV polarization is found to be more suitable for soil moisture retrieval compared to VH polarization, with an R^2^ of 0.672 and an RMSE of 0.048m^3^·m^-3^, the accuracy is higher than that of the direct retrieval method. The results of the study preliminarily verified the feasibility of the coupling method in soil moisture retrieval over densely veg etated surfaces.

## Introduction

Soil moisture is a crucial medium for the exchange of matter and energy within the Earth’s ecosystem, and plays a pivotal role in processes such as water cycle, climate change, and ecological evolution [1]. In the field of agricultural production, soil moisture has direct impact on the development of crop root systems, photosynthesis, and biomass accumulation [2,3]. Timely and accurate soil moisture monitoring is of great practical significance for agricultural drought forecasting, water resource management and crop yield estimation.

Traditional soil moisture measurement methods, such as the gravimetric method and the Time Domain Reflectometry (TDR) method are conducted at a point scale, which requires substantial human and material resources and cannot meet the demands of dynamic and large-scale soil moisture monitoring. With the rapid development of remote sensing technology, the estimation of soil moisture with high spatiotemporal resolution is becoming increasingly practical. Based on the type of sensors carried by satellites, remote sensing can be broadly categorized into optical remote sensing and microwave remote sensing [4]. For optical remote sensing, several indices that utilize visible-near-infrared, hyperspectral, and thermal infrared bands have been proposed for soil moisture monitoring, such as the Shortwave-infrared Perpendicular Drought Index (SPDI) [5], the Distance Drought Index (DDI) [6], the Visible and Shortwave-infrared Drought Index (VSDI) [7] and the Triangle Soil Moisture Index (TSMI) [8]. However, these indices just establish an empirical relationship between spectral bands and soil moisture, lacking physical foundations [9]. Additionally, optical remote sensing are susceptible to meteorological factors such as clouds and rain, which limits its potential application.

Microwave remote sensing, on the other hand, has the advantage of all-weather, all-day observations and it is physically correlated with soil moisture. Among various microwave remote sensing datasets, synthetic aperture radar (SAR) remote sensing has emerged as the most promising method for soil moisture observation due to its high sensitivity to soil dielectric constant and high spatio-temporal resolution [10,11]. Over bare surfaces, soil moisture can be effectively estimated using SAR remote sensing data with either theoretical models (e.g., the Integral Equation Model (IEM) [12], the Advanced Integral Equation Model (AIEM) [13]) or statistical models (e.g.,the Oh model [14], the Dubois model [15]). Ezzahar et al. experimentally verified the feasibility of using SAR backscattering data to invert surface soil moisture over bare surfaces [16]. Santi et al. introduced machine learning for estimating soil moisture with C-Band SAR data [17]. Baghdadi et al. estimated soil moisture over bare agriculture areas with C-band polarimetric SAR data using neural networks, and achieved good accuracy [18].

Although SAR remote sensing can perform well in soil moisture retrieval over bare surface, it is not so effective over vegetated areas, since the sensitivity of SAR signals to soil moisture decreases due to the absorption and scattering effects of the vegetation layer [19,20]. The Water Cloud Model (WCM) is the most widely used model for distinguishing the SAR backscattering contributions from the vegetation layer and soil layer over agricultural fields, and the vegetation water content (VWC) was specifically defined in the WCM to calculate the direct contribution and attenuation of vegetation layer [21]. Thus, making accurate estimation of VWC is of primary importance for improving the performance of the WCM and, consequently, for enhancing the precision of soil moisture retrieval.

Previous studies have indicated that the VWC is closely related to the normalized differential vegetation index (NDVI), which can be derived from optical remote sensing images [22,23]. Baghdadi et al developed an inversion approach for estimating surface soil moisture of crop fields and grasslands with the WCM and the NDVI using Sentinel-1/2 data [24]. El Hajj compared the soil moisture estimation potentials of the C-band and L-bands SAR for wheat and grassland plots by using the NDVI and the WCM [25]. The coupling of SAR and optical remote sensing has been considered to be an effective way for soil moisture retrieval over vegetated areas [26–29]. However, most of the existing studies focus on soil moisture retrieval over low vegetation covered regions, such as grassland, winter wheat and rice [30,31]. With the increase of vegetation height and coverage, the interaction between SAR signals and vegetation becomes more complicated, and the NDVI derived from optical remote sensing imaged tends to be saturated, which is easy to be neglected by researchers [32,33]. Therefore, for surfaces with tall and dense vegetation cover, a single vegetation index is not inadequate for estimating VWC in the WCM, and the accuracy and applicability of the cooperative approach of SAR and optical remote sensing in soil moisture retrieval remain to be explored over dense vegetated areas.

In this study, a typical agricultural area in the Nanyang Basin was selected to conduct field observation experiments, where the majority of the surfaces is covered by dense summer maize at the mid-late growth stage. Using Sentinel-1A SAR data and Sentinel-2 Multi Spectral Instrument (MSI) data, an coupling inversion approach of soil moisture over densely vegetated surfaces was explored. To address the problem of decreased sensitivity between SAR signals and soil moisture due to the dense vegetation layer, the relationship between nine optical vegetation indices and measured VWC was systematically analyzed, and the most well-performed vegetation indices were selected to establish VWC estimation model, which was combined with the WCM to eliminate the influence of the vegetation layer on SAR backscattering coefficients. On this basis, the regression analysis method, and the look-up table (LUT) algorithm are respectively adopted for soil moisture retrieval, and the soil moisture retrieval accuracy with different methods and SAR polarization modes were compared. The research aims to provide scientific supports for obtaining soil moisture information from SAR and optical remote sensing imagery over dense vegetated surfaces.

## Study area and data description

### Study area

**Fig 1:**
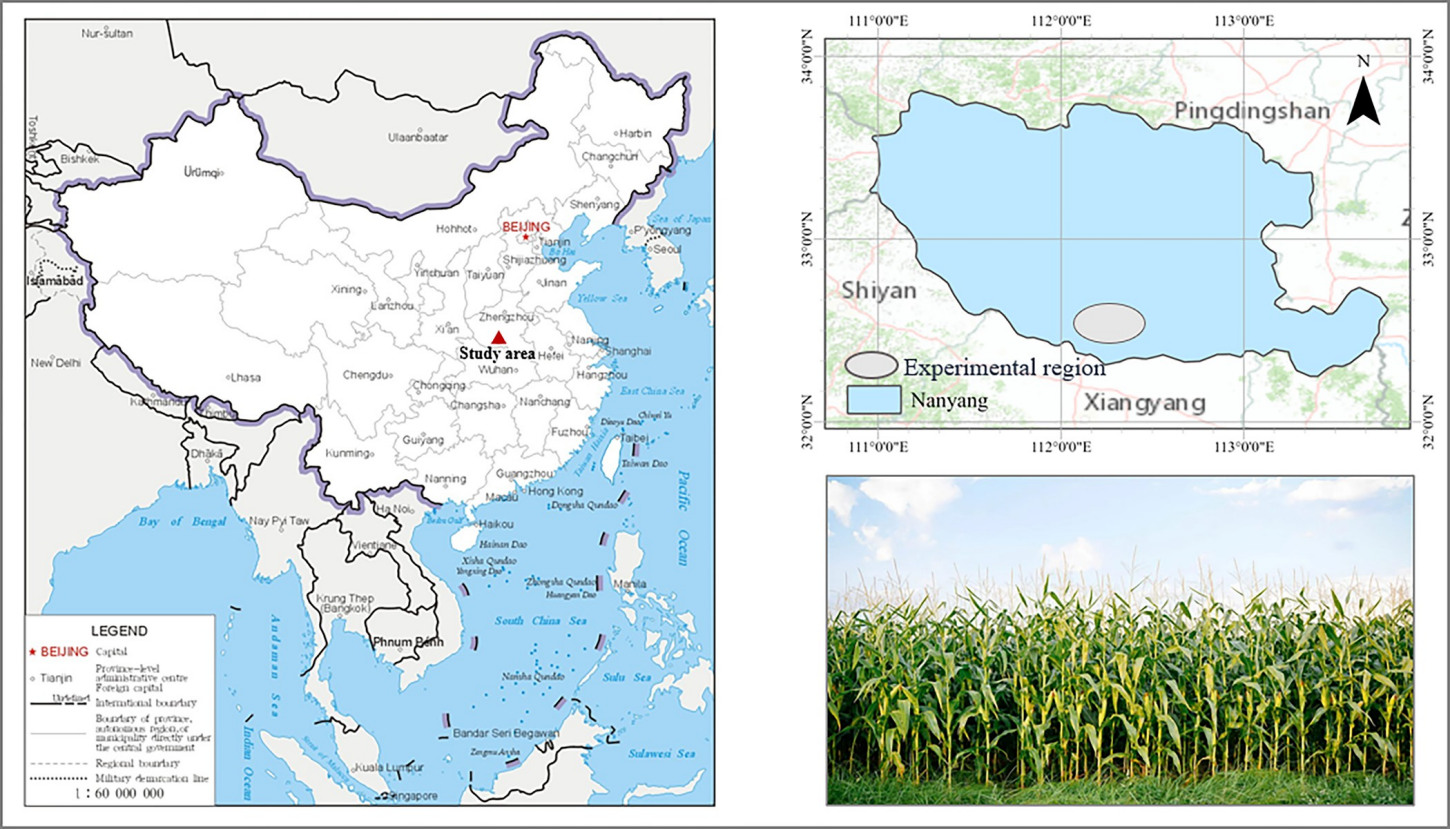
Location of the study area and the experimental region.

The study area is located in Nanyang City, Henan Province (32°17′~33°48′ N, 110°58′~113°49′ E), which has a warm-temperate continental monsoon climate with abundant sunlight and distinct seasons. The mean annual temperature is 15°C, and the average annual precipitation is about 800 mm. The region has a flat topography with predominantly yellow cinnamon soil and yellow brown loam soil. The prevalent cropping system follows a winter wheat-summer maize rotation, with summer maize usually planted in late June and harvested in early October. During the ground experiment, the summer maize was mostly in the tasseling stage, with an average plant height of approximately 1.7 m and a mean canopy coverage exceeding 80%. Fig 1 shows the location of the study area and the experimental region.

The shapefile is reprinted from Map Technical Review Center, Ministry of Natural Resources of China under a CC BY license, with permission from Ministry of Natural Resources of China (http://bzdt.ch.mnr.gov.cn). The figure was made with ArcGIS pro under a CC BY license, with permission from ESRI (www.esri.com).

### SAR and optical remote sensing data

The Ground Range Detected (GRD) Level-1 images from the Sentinel-1A SAR satellite, acquired in interferometric wide swath mode was used in this study. The satellite is equipped with the C-band SAR sensor, operating at a frequency of 5.4 GHz, and captures data in two polarization modes: VV (vertical transmit and vertical receive) and VH (vertical transmit and horizontal receive). The spatial resolution of the images is 5 m × 20 m, with a swath width of 250 km. The SAR imagery was acquired on August 8, 2023. The pre-processing of the SAR data involves essential steps such as orbit correction, radiometric calibration, filtering, and terrain correction. These procedures are conducted using the Sentinel Application Platform (SNAP) provided by the European Space Agency (ESA). After pre-processing, Eq (1) is applied to convert the grayscale values of SAR images into backscattering coefficients:

$$\sigma_{i,j}^{0}(\theta )=10lg\frac{{DN}_{i,j}^{2}}{A_{\sigma }^{2}}$$ (1)

where $\sigma_{i,j}^{0}\;(\theta )$ is the backscattering coefficient of the element in the i th row and j th column of the SAR image at the incidence angle *θ*, dB; *DN*_*i*,*j*_ is the gray value of the pixel in the i th row and j th column of the SAR image; $A_{\sigma }^{2}$ is the calibration parameter.

The optical remote sensing data used in the study is the Level-L2A level atmospheric bottom reflectance products of Sentinel-2 MSI imagery, which have undergone radiometric calibration and atmospheric correction. Sentinel-2 MSI imagery contains a total of 11 spectral bands with three different spatial resolutions of 10m, 20m, and 60m. According to the acquisition date of Sentinel-1 SAR imagery and cloud cover, the quasi-synchronous Sentinel-2 MSI image was acquired on August 5, 2023. To facilitate the extraction and analysis of the spectral information, SNAP was used in advance to resample the low-resolution bands to high resolution.

### Ground observation

**Fig 2:**
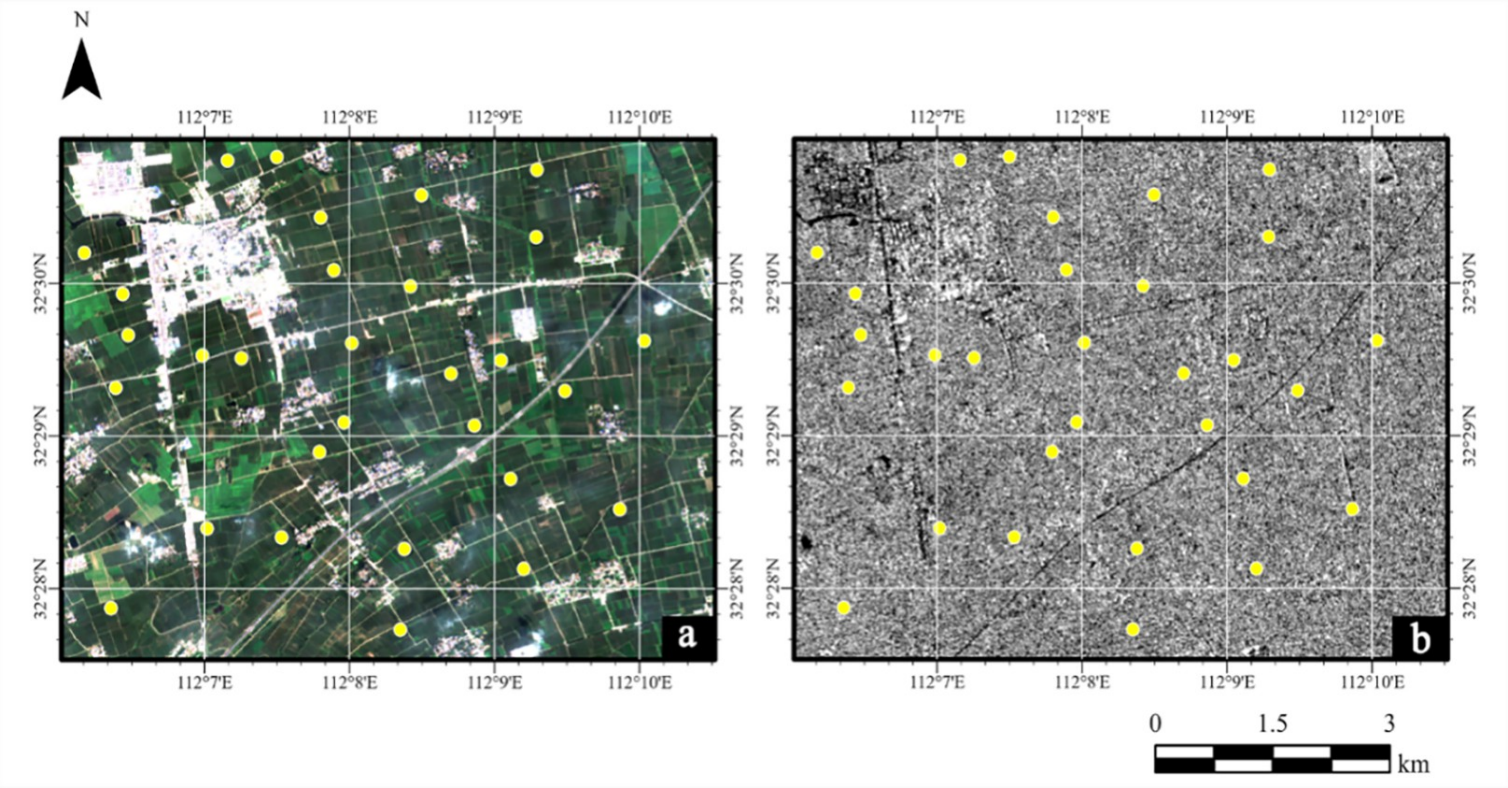
Distribution of sampling sites (a. Sentinel-2 imagery in RGB band (R = band; G = band; B = band; b. Sentinel-1 imagery in VV polarization). Sentinel-1 and Sentinel-2 images are made freely available to the general public by the European Space Agency (ESA) (https://dataspace.copernicus.eu/).

The ground observation experiment was conducted synchronously with the transit date of Sentinel-1 SAR satellite. Measured data of a total of 32 sampling points was collected in the observational experiment. To further mitigate the impact of mixed pixels on the inversion results, the location of sampling point should adhere to the following principles: 1) at a single crop type covered area, and 2) the distance between the two adjacent sampling points, as well as the distance between each sampling point and other features such as roads and rivers, should be at least 20 meters. Two sampling points affected by cloud cover in the Sentinel-2 MSI imagery were excluded, and the soil moisture inversion was conducted using the measured data from the remaining 30 sampling points. The distribution of sampling points is illustrated in Fig 2.

**Fig 3:**
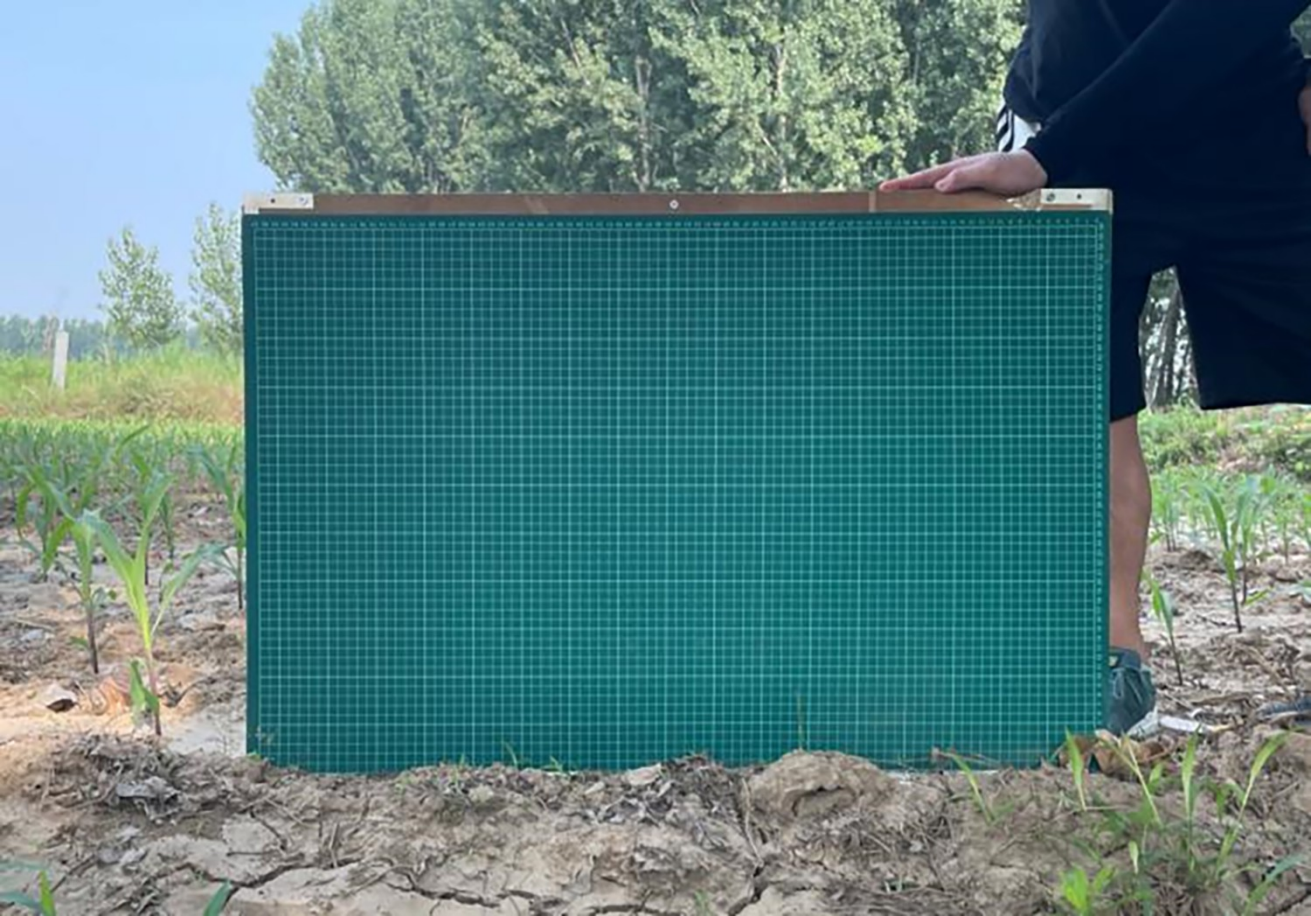
Surface roughness parameters measurement.

Ground observation data include the soil moisture, surface roughness, VWC and the latitude and longitude at each sampling point. Considering the penetration of C-band Sentinel-1 SAR sensor, soil moisture was measured using a portable TDR 300 soil moisture meter at depths of 7.5 cm. Within a 10-meter radius around each sampling point, five measurement points were uniformly selected for soil moisture measurements. The average of these five measurements was used as the soil moisture value for that sampling point to mitigate the impact of spatial heterogeneity on measurement results. The surface roughness was determined by the roughness plate method at the early stage of maize growth. A roughness calibration plate with 0.9m in length and 0.6m in width was placed vertically on the target surface, surface contour information was captured by a digital camera, and the surface roughness parameters including surface root-mean-square height (RMS) and the surface correlation length (L) were extracted (Fig 3). The measured values of the RMS of the surface in the 32 sample points ranged from 1.42 cm to 1.55 cm, and the measured values of the L ranged from 14.3 cm to 18.1 cm, which are in line with the surface roughness characteristics of the cultivated land with little spatial variability. VWC was determined by the drying method. Firstly, plant samples from the summer maize at each sampling point were collected, the fresh weight of the plant samples was measured instantly using the electronic balance, then the plant samples were dried in the oven to obtain the dry weight. VWC was calculated using the fresh weight and the dry weight of plant samples. A portable dual-frequency global positioning system (GPS) receiver was used to record latitude and longitude information of each sampling site with centimeter-level accuracy.

## Methods

### WCM

**Fig 4:**
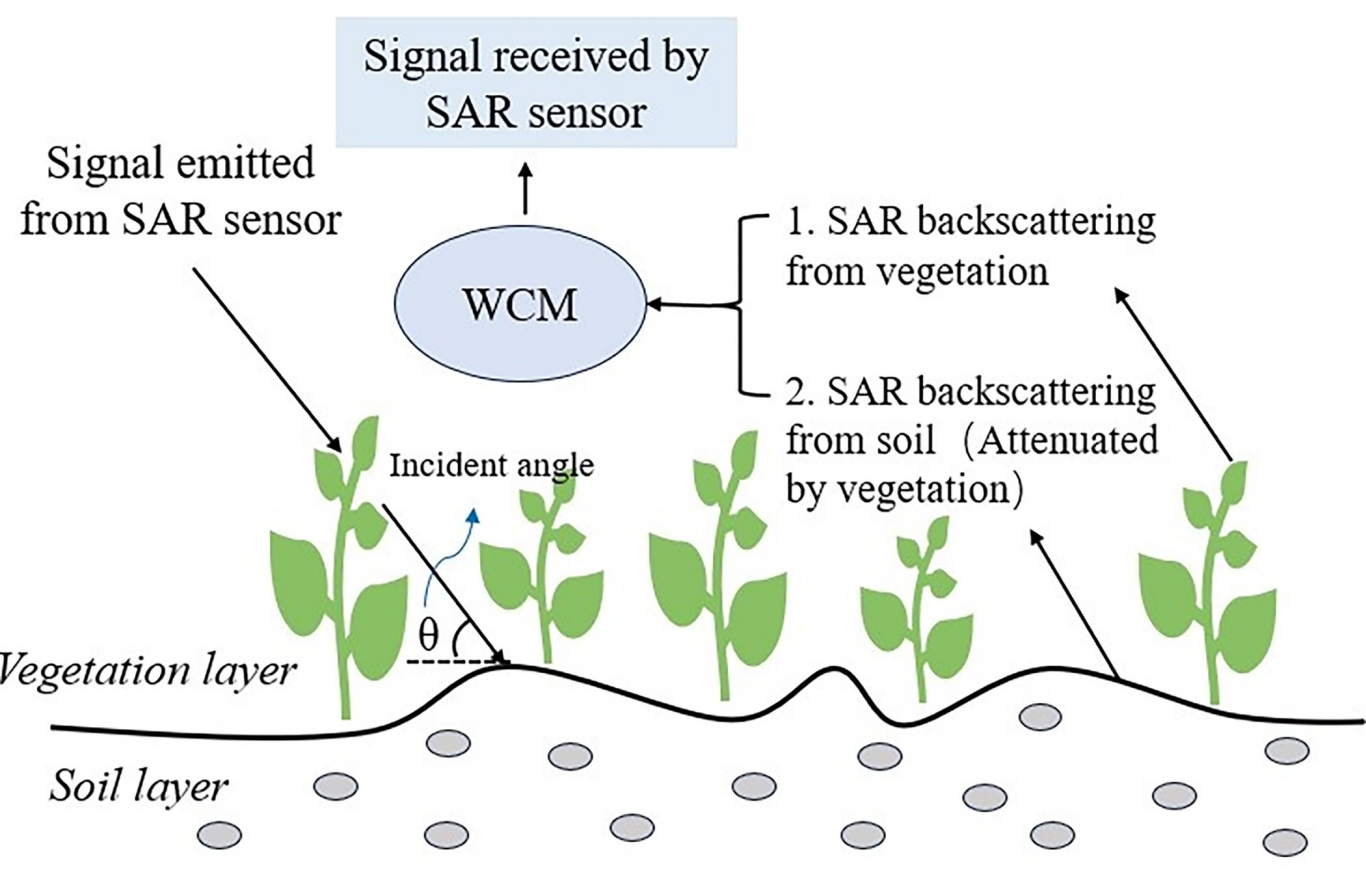
Conceptual scattering of SAR signal over vegetation covered surfaces described by WCM.

In the 1970s, Attema and Ulaby proposed the WCM based on the radiative transfer equation by analyzing the microwave scattering mechanism from vegetation-covered surfaces [34]. The model assumes the vegetation layer as an isotropic homogeneous scatterer, i.e., a "cloud layer", and ignores the multiple scattering processes between the vegetation and the surface, and the SAR backscattering coefficient of the vegetation-covered surface is determined by the body scattering from the vegetation itself and the surface scattering after double attenuation by the vegetation layer (Fig 4).

The specific representation of the WCM is shown in Eqs (2)–(4).

$$\sigma^{0}=\sigma_{veg}^{0}+\tau^{2}\sigma_{soil}^{0}$$ (2)

$$\sigma_{veg}^{0}=A{\cdot M}_{veg}\cdot cos(\theta )(1-\tau^{2})$$ (3)

$$\tau^{2}=exp[-2B{\cdot M}_{veg}\cdot \sec\left(\theta \right)]$$ (4)

where *σ*^0^ is the total backscattering coefficient of the vegetation-covered surface, dB; $\sigma_{veg}^{0}$ is the direct backscattering coefficient of the vegetation layer, dB; $\sigma_{soil}^{0}$ is the direct backscattering coefficient of the soil layer, dB; *τ*^2^ is the double-layer attenuation factor of the vegetation layer; *θ* is the incident angle of the SAR sensor, rad; *M*_*veg*_ is the water content of the vegetation, kg·m^-2^; and *A* and *B* are empirical parameters depending on the type of the vegetation and the frequency of the electromagnetic wave.

### VWC estimation model

VWC is a key factor affecting the intensity of microwave backscattering from the vegetation layer, it is also a crucial parameter in the WCM. Based on the spectral reflectance information from Sentinel-2 MSI remote sensing data, nine vegetation indices sensitive to the moisture content in plant canopy were extracted by band calculation, including the Simple Ratio Vegetation Index (SR), NDVI, the Normalized Difference Water Index (NDWI), the other form the Normalized Difference Water Index (NDWI2), the Modified Normalized Difference Water Index (MNDWI), the Moisture Stress Index (MSI), the Normalized Difference Greenness Index (NDGI), the Fusion Vegetation Index (FVI), and the Enhanced Vegetation Index (EVI). The equations of different vegetation indices are shown in Table 1.

**Table 1:** Formulas of optical vegetation index.

Vegetation index	Full name	Formula	Reference
SR	Simple Ratio Vegetation Index	NIR/R (5)	[35]
NDVI	Normalized Difference Vegetation Index	(NIR−R)/(NIR+R) (6)	[36]
NDWI	Normalized Difference Water Index	(G−NIR)/(G+NIR) (7)	[37]
NDWI2	Normalized Difference Water Index 2	(NIR−SWIR)/(NIR+SWIR) (8)	[38]
MNDWI	Modified Normalized Difference Water Index	(G−SWIR)/(G+SWIR) (9)	[39]
MSI	Moisture Stress Index	SWIR/NIR (10)	[40]
NDGI	Normalized Difference Greenness Index	(G−R)/(G+R) (11)	[41]
FVI	Fusion Vegetation Index	(2NIR−R−SWIR)/(2NIR+R+SWIR) (12)	[42]
EVI	Enhanced Vegetation Index	2.5(NIR−R)/(NIR+6R−7.5B+1) (13)	[43]

Two modeling approaches were employed in the study to establish the VWC estimation model. One approach involved establishing the relationship between a single vegetation index and the measured VWC, with a single-factor regression analysis method. While the other approach further utilized the advantages of different optical spectral bands, focused on establishing the relationship between multiple vegetation indices and the measured VWC. To be more specific, by comparing the nine VWC estimation models constructed based on different vegetation indices, the most well-performed vegetation indices were selected to develop the VWC estimation model using a multi-factor regression analysis method. Finally, the VWC estimation model with the best accuracy was then employed in the subsequent vegetation correction. For both single-factor regression method and multi-factor regression method, 70% of the measured data was selected randomly to establish the VWC estimation models, and the other 30% data was used to verify the accuracy of these models.

### Oh model

After correcting for vegetation effects using the VWC estimation model and the WCM, the obtained direct surface backscattering coefficients are not only closely related to soil moisture, but also affected by the SAR sensor parameters, surface roughness, soil texture and so on. To accurately establish the relationship between soil moisture and backscattering coefficient, the influence of other factors must be taken into account. Oh and Sarabandi analyzed the relationships between SAR co-polarization ratio, cross-polarization ratio, surface roughness, and soil dielectric constant using a large amount of field measurement data [44–46]. After multiple refinements and improvements, the semi-empirical Oh model was proposed for establishing the relationship between soil moisture and SAR backscattering coefficient over bare surfaces. The Oh model is characterized by its simplicity and wide range of applications, and has been generally recognized in the field of soil moisture retrieval [16,47]. The expressions are as follows:

$$\sigma_{vh}^{0}=0.11m_{v}^{0.7}{\left(\cos\theta \right)}^{2.2}[1-exp{\left(-0.32ks\right)}^{1.8}]$$ (14)

$$q=\frac{\sigma_{vh}^{0}}{\sigma_{vv}^{0}}=0.1{\left[\frac{s}{l}+sin\;(1.3\theta )\right]}^{1.2}\{1-\exp[-0.9{\left(ks\right)}^{0.8}]\}$$ (15)

where $\sigma_{vv}^{0}$ and $\sigma_{vh}^{0}$ are the backward scattering coefficients of the bare ground surface under VV and VH polarization, respectively, dB; s is the root-mean-square height, cm; l is the correlation length, cm; and k is the free-space wave number of the electromagnetic wave.

### Accuracy verification

Coefficient of determination (R^2^) and root mean squared error (RMSE) are used as accuracy evaluation indices, the calculation formulas are shown in Eqs (16) and (17). A higher R^2^, closer to 1, and a smaller RMSE indicate a higher accuracy of the model.

$$R^{2}=1-\frac{\sum_{i=1}^{n}\;(y_{i}-{\hat{y}}_{i}{)}^{2}}{\sum_{i=1}^{n}\;(y_{i}-\bar{y}{)}^{2}}$$ (16)

$$RMSE=\sqrt{\frac{\sum_{i=1}^{n}\;({\hat{y}}_{i}-y_{i}{)}^{2}}{n}}$$ (17)

where ${\hat{y}}_{i}$ is the predicted value of soil moisture at each sampling point, m^3^·m^-3^; $\bar{y}$ is the mean value of soil moisture at each sampling point, m^3^·m^-3^; *y*_*i*_ is the measured value of soil moisture at each sampling point, m^3^·m^-3^; and *n* is the number of samples.

### Soil moisture cooperative retrieval methods

**Fig 5:**
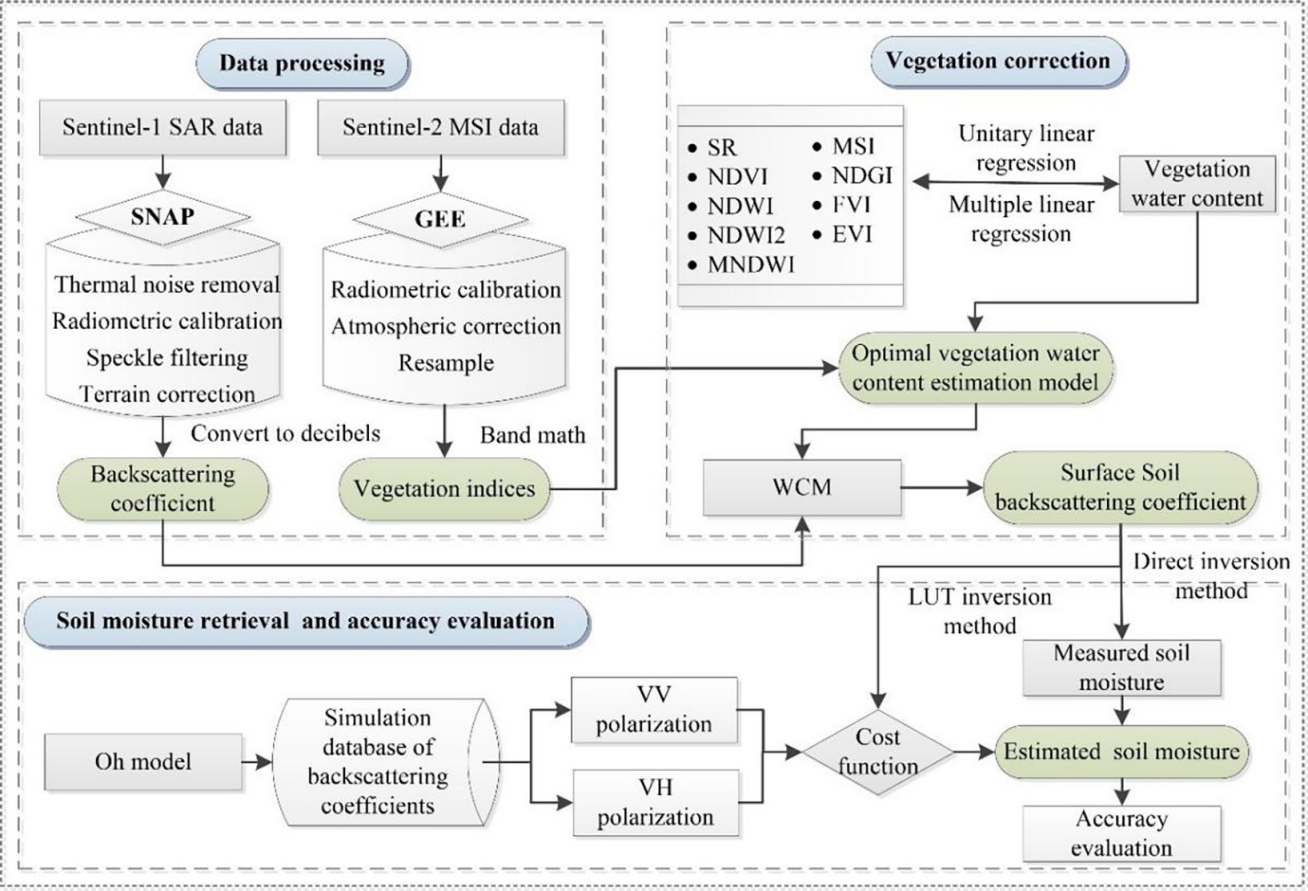
The technology roadmap.

The specific steps of the soil moisture cooperative retrieval approach are as follows: (1) In the Google Earth Engine (GEE), Sentinel-2 MSI remote sensing data is used to extract nine vegetation indices including SR, NDVI, NDWI, NDWI2, MNDWI, MSI, NDGI, FVI and EVI. Relationships are established between each vegetation index and the measured VWC, then the optimal vegetation indices are selected to build the multi-factor regression model for VWC estimation. (2) The VWC estimation model is combined with WCM to eliminate the influence of summer maize vegetation on SAR backscattering coefficients, and the direct backscattering coefficient of the soil layer is obtained. (3) The Oh model is employed to simulate the relationship between soil moisture and soil layer direct backscattering coefficients. A simulation database of backscattering coefficients under different polarization modes is constructed. (4) The direct retrieval method and the LUT method are used for soil moisture retrieval, respectively, and the impact of retrieval methods, as well as polarization modes on the accuracy of soil moisture inversion is investigated using ground-measured soil moisture data. The specific technical route is shown in Fig 5.

## Results and discussion

### VWC estimation model

#### Correlation analysis

**Fig 6:**
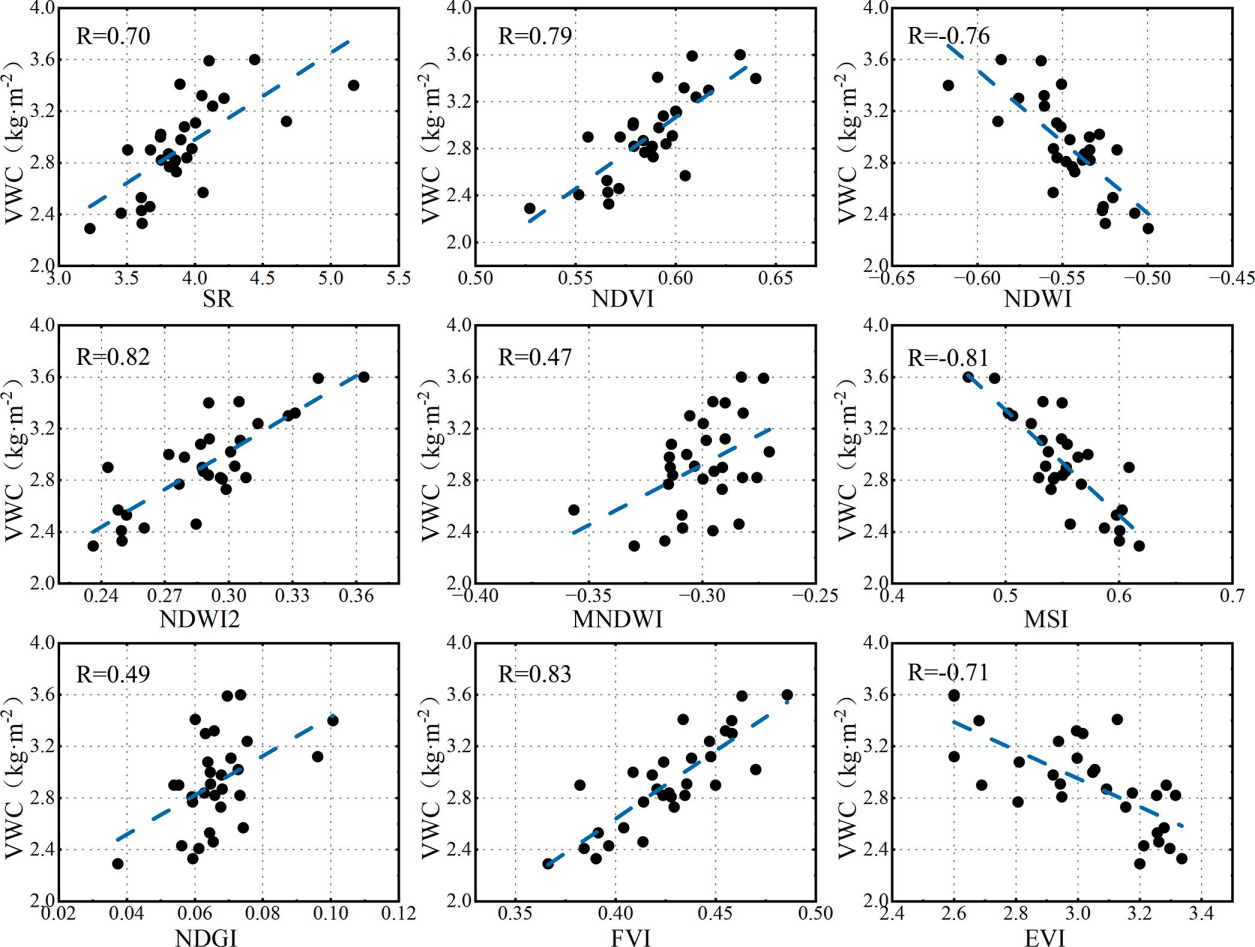
Correlation relationships between different vegetation indices and VWC.

Fig 6 shows the correlation coefficient (R) between each vegetation index and measured VWC. Results show that among the nine vegetation indices, SR、NDVI、NDWI2、MNDWI、NDGI and FVI are positively correlated with VWC, while NDWI、MSI and EVI have negative correlation with VWC. The vegetation index with the highest correlation was FVI (R = 0.83), followed by NDWI2(R = 0.82) and MSI(R = -0.81), while NDGI and MNDWI have poor correlation relationships with VWC, the R of which were 0.49 and 0.47, respectively.

#### Regression models for VWC estimation

**Fig 7:**
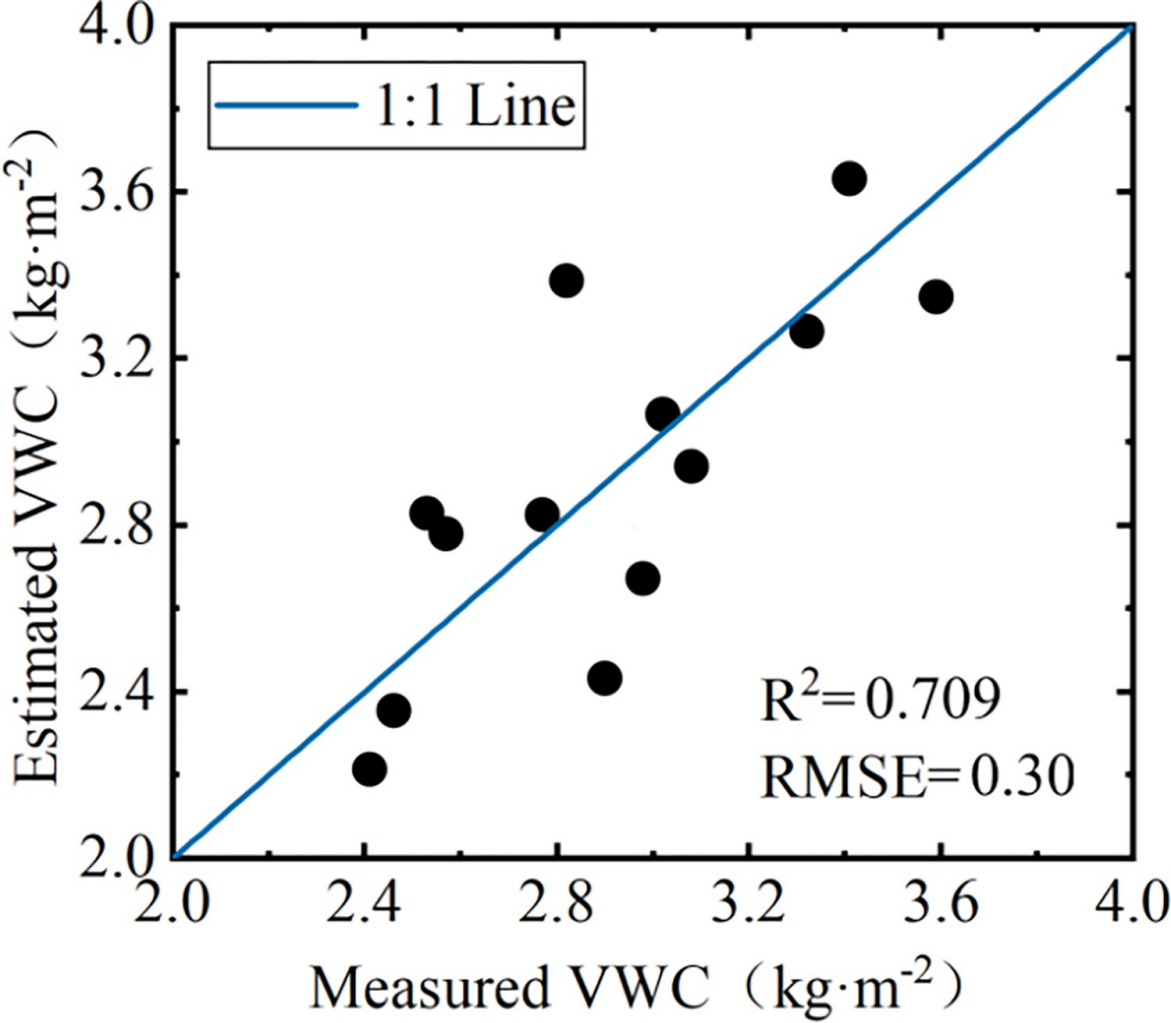
Estimation accuracy of VWC with the multiple regression model.

The single-factor regression analysis method and the multi-factor regression analysis method were used for VWC estimation. For both methods, the measured data were randomly divided into two groups according to the ratio of 6:4, the former is used for establishing estimation models, and the latter is used for accuracy verification. The VWC estimation models and the accuracy are shown in Table 2 and Fig 7.

**Table 2:** Formulas and accuracies of the VWC estimation models based on different vegetation indices.

Vegetation index	Fitting formula	R^2^	RMSE (kg·m^-2^)
SR	y = 0.6736x + 0.2849	0.480	0.362
NDVI	y = 12.16x - 4.2289	0.621	0.325
NDWI	y = -11.076x - 3.1282	0.212	0.419
NDWI2	y = 9.7698x + 0.0931	0.667	0.311
MNDWI	y = 9.2867x + 5.7056	0.217	0.423
MSI	y = -8.1415x + 7.414	0.485	0.363
NDGI	y = 15.241x + 1.907	0.664	0.312
FVI	y = 10.53x - 1.5734	0.693	0.303
EVI	y = -1.0938x + 6.2326	0.503	0.358

The single-factor regression results suggested that FVI is the most well-performed vegetation index for VWC estimation, with an R^2^ of 0.693 and RMSE of 0.303 kg·m^-2^. NDVI, NDWI2 and NDGI also show good linear relationships with VWC, the R^2^ between NDVI, NDWI2, NDGI and summer maize VWC are 0.621, 0.655, 0.693, the RMSE are 0.324 kg·m^-2^, 0.311 kg·m^-2^ and 0.312 kg·m^-2^, respectively. While the sensitivity of SR, NDWI, MNDWI and MSI to VWC of summer maize was relatively poor, with R^2^ of 0.480, 0.212, 0.217, 0.485 and RMSE of 0.362 kg·m^-2^, 0.419kg·m^-2^, 0.423kg·m^-2^, 0.358kg·m^-2^. According to the formula of each vegetation index, it can be seen that compared with other bands, the combination of near-infrared band and short-wave infrared band has advantages in the estimation of vegetation water content in dense vegetation cover area. This is mainly because water molecules exhibit strong reflective characteristics in the near-infrared band and strong absorption characteristics in the short-wave infrared band, vegetation indices which incorporate these two spectral bands effectively strengthen the connection between spectral features and VWC [48].

Further, to integrate the advantages of different vegetation indices, a multiple regression model was established with the four best-performing vegetation indices, namely NDVI, NDWI2, NDGI and FVI, as independent variables and VWC as the dependent variable. The model was expressed in Eq (18).


VWC=5.9803NDVI+3.0716NDWI2−2.4564NDGI+4.1985FVI−3.1130 (18)

Fig 7 illustrates the estimation accuracy of VWC with the multiple regression model with the validation dataset. Results indicate that the estimated values of VWC are close to the actual measurements, the majority of sample points are distributed in the vicinity of the 1:1 line, and it is noticed that when the VWC is below 2.5 kg·m^-2^, the estimated value of VWC exhibit a general underestimation compared to the actual measurements. Compared to models that constructed with a single vegetation index, the accuracy of multiple regression model was improved, with an R^2^ of 0.709, and an RMSE of 0.30 kg·m^-2^. Therefore, the multiple regression model is more effective in estimating the VWC of summer maize, and can be applied in the subsequent correction of vegetation effects.

### Vegetation correction

**Fig 8:**
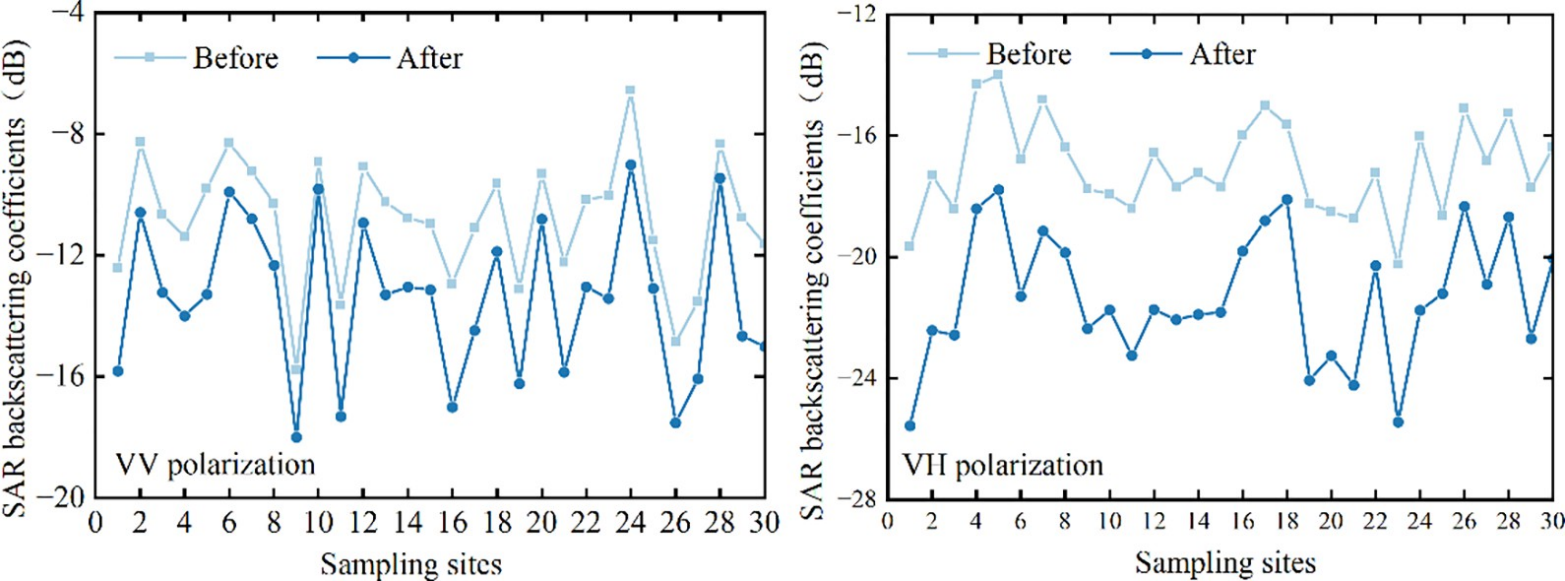
Comparison of microwave backscatter coefficients before and after vegetation correction.

The microwave backscattering coefficients obtained from Sentinel-1 SAR images, and the VWC estimated from the multiple regression model were input into the WCM to eliminate the absorption and scattering effects of the vegetation layer on SAR backscattering signals. For different SAR polarization modes, the microwave backscattering coefficients of each sampling point before and after vegetation correction are shown in Fig 8.

The results suggested that the microwave backscattering coefficients in the co-polarization and cross-polarization modes show different degrees of decreases after remove the influence of vegetation layer. For VV polarization mode, the backscattering coefficients decreased from -15.77 ~ -6.56 dB to -18.02 ~ -9.01 dB, with an average decrease of about 3 dB. For VH polarization mode, the backscattering coefficients decreased from -20.24 ~ -14.81 dB to -25.56 ~ -17.77 dB, with an average decrease of about 4 dB. The extent of the reduction depends mainly on the VWC and the total SAR backscattering coefficient. In addition, since SAR co-polarization is more sensitive to dihedral angular reflectance effects, the backscattering coefficient is usually higher in the VV polarization mode than in the VH polarization mode.

**Fig 9:**
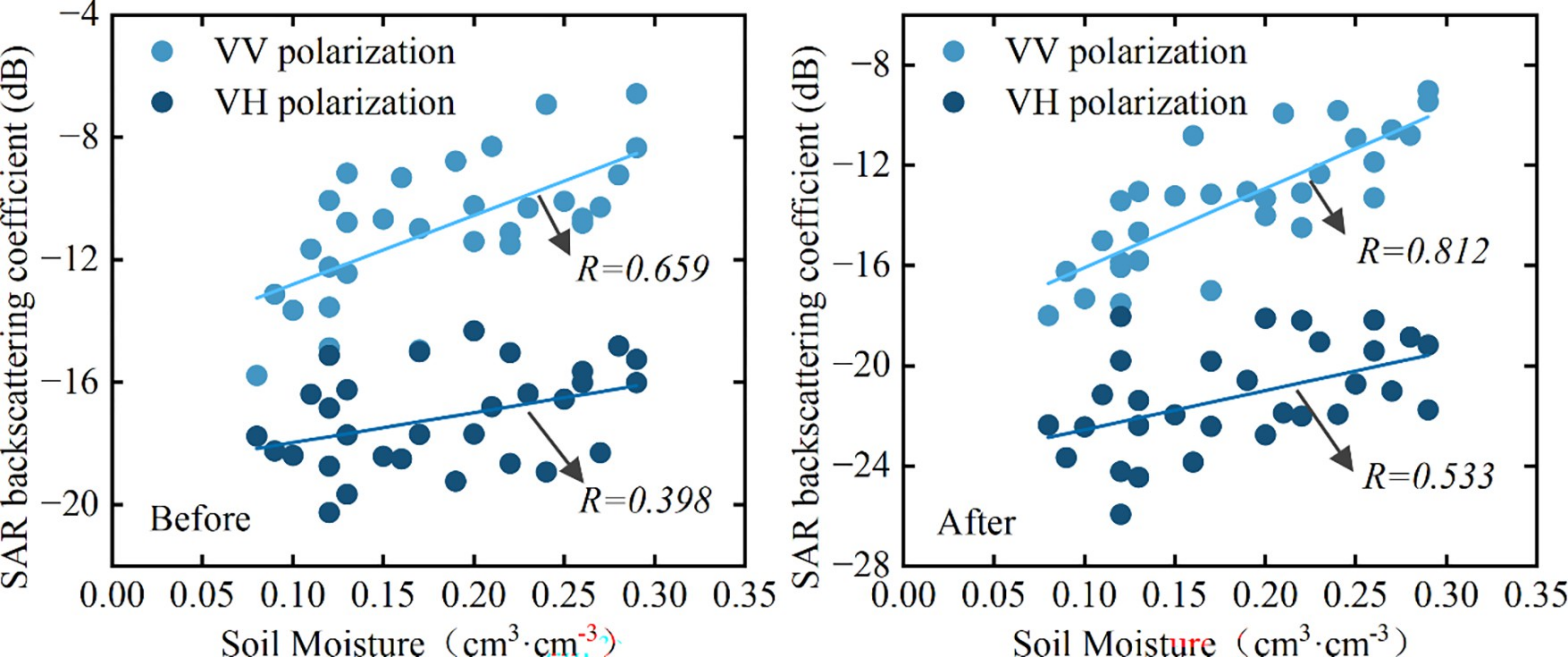
Scatter diagram of SAR backscattering coefficient to soil moisture before and after vegetation correction.

Pearson correlation analysis was used to analyze the correlation between SAR backscattering coefficients and soil moisture before and after vegetation correction, and the results are shown in Fig 9. It can be seen that there is an obvious positive correlation between SAR backscattering coefficients and soil moisture, and the correlation in the VV polarization mode is more significant compared to VH polarization mode. After eliminating the effects of the vegetation layer on SAR backscattering signals, the correlations between backscattering coefficients and soil moisture under different polarization modes were both improved. The Pearson’s correlation coefficient increased from 0.659 to 0.802 for the VV polarization mode, and from 0.398 to 0.509 for the VH polarization mode. The results suggested that the combination of the VWC estimation model and the WCM can effectively identify the contribution of the vegetation layer in the SAR backscattering coefficient, this enables obtaining the direct backscattering coefficient of soil layer under dense vegetation cover, providing support for improving the accuracy of the soil moisture retrieval.

### Soil moisture retrieval

The backscattering coefficient of the soil layer, obtained after vegetation correction, is a function of multiple variables such as soil moisture, SAR sensor parameters, surface roughness, and soil texture. Some researchers conduct soil moisture retrieval by directly establishing a regression model between soil layer backscattering coefficients and soil moisture. Although the method is straightforward and easy to operate, it is unable to eliminate the interference from parameters other than soil moisture in SAR backscattering signals [49]. Therefore, in this study, a more comprehensive LUT method was employed for soil moisture retrieval based on the microwave scattering model-Oh model, which considers the effects of soil moisture, SAR incidence angle, and surface roughness parameters on the backscattering coefficients, thus effectively reducing the uncertainty in soil moisture retrieval. In addition, the direct retrieval method was also investigated and employed in the study for comparative purpose.

#### Direct retrieval method

**Fig 10:**
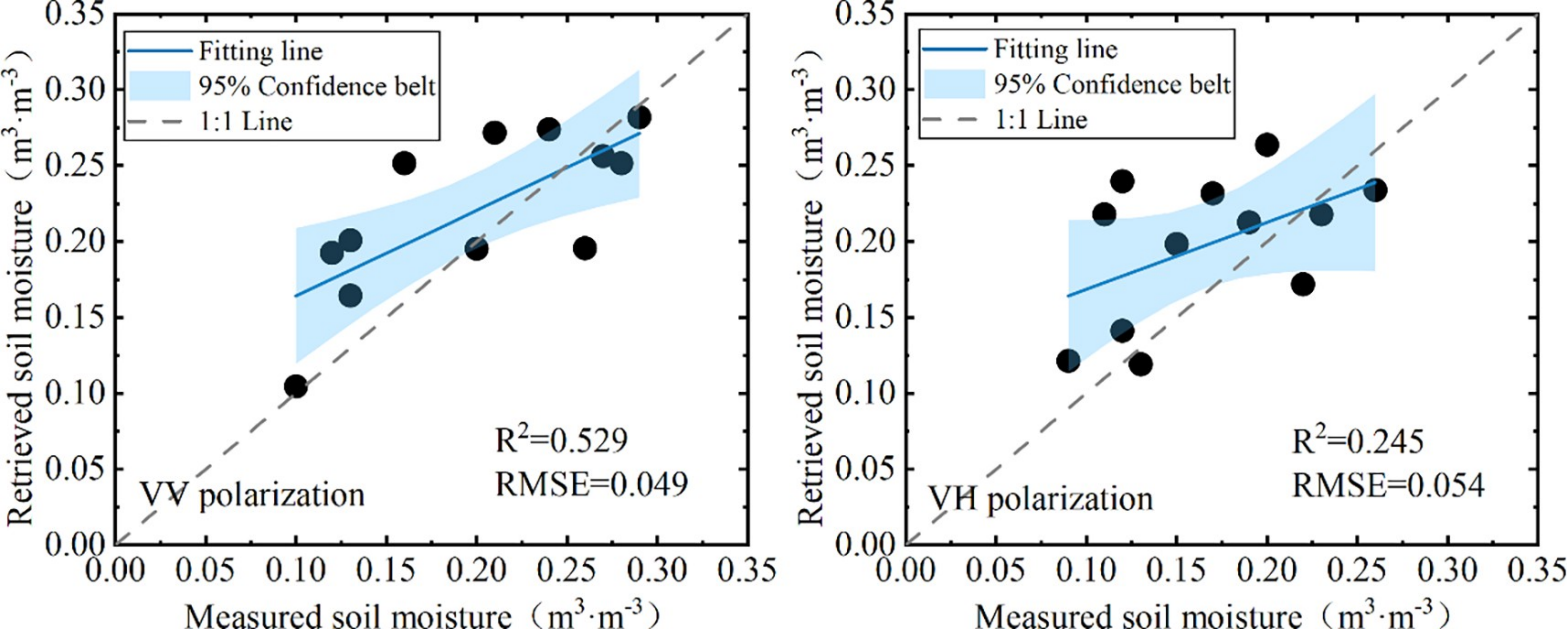
Performance of the direct retrieval method in soil moisture retrieval under different SAR polarization modes. (a. VV polarization; b. VH polarization).

Based on measured data, the direct retrieval method retrieves soil moisture by establishing a fitting relationship between soil moisture and the backscattering coefficient of soil layer. The measured data were initially randomly split into a training dataset and a validation dataset in a 6:4 ratio. The training dataset was used to establish soil moisture retrieval model, and the validation dataset was employed to evaluate the accuracy of the model. Fig 10 illustrates the performance of the direct retrieval method in soil moisture retrieval at different SAR polarization modes. Results suggest that the R^2^ between the retrieved soil moisture and the measured value is 0.529, and the RMSE is 0.049 m^3^·m^-3^ at VV polarization mode. For VH polarization mode, the R^2^ between the retrieved soil moisture and the measured value is 0.245, and the RMSE is 0.054 m^3^·m^-3^. It can be seen from the results that, without considering the impact of other surface parameters on the backscattering coefficient of soil layers, the model established by the direct retrieval method could provide a rough estimate of soil moisture, but the accuracy needs to be improved. Besides, the retrieval accuracy of soil moisture at VV polarization is significantly higher than that at VH polarization.

#### LUT method

LUT method is one of the most widely used retrieval techniques in the field of quantitative remote sensing, it uses a large amount of simulated data to establish the relationship between the space of parameters to be retrieved and the space of observations, and to create mappings in the space of observations with certain similarities. Every time a new set of observation data is obtained, the most similar record can be found in the LUT through the cost function, and the parameter to be retrieved can be read out in that record. In this study, the simulation database generated by the Oh model was used to establish the LUT between the parameters to be retrieved and the observed data. For an observed data, a metric relationship with a certain similarity is established, when a new set of observation data is obtained, the most similar record can be found in the LUT through the cost function, and the parameter to be retrieved can be read out in the record. Matlab R2022a was used to simulate backscattering coefficients of the soil layer corresponding to different surface parameters including SAR incidence angle, soil moisture, root mean square height, and correlation length, as well as establish the simulation database. According to the measured surface data, the input parameters and step size of the Oh model is determined in Table 3.

**Table 3:** Input parameters and step size of the backscattering coefficients simulation database.

Input parameter	Unit	Minimum value	Maximum values	Step size
Incidence angle	°	30	40	2
Soil moisture	cm^3^·cm^-3^	0.05	0.35	0.01
Root mean square height	cm	0.6	2.0	0.2
Correlation length	cm	4	20	2

**Fig 11:**
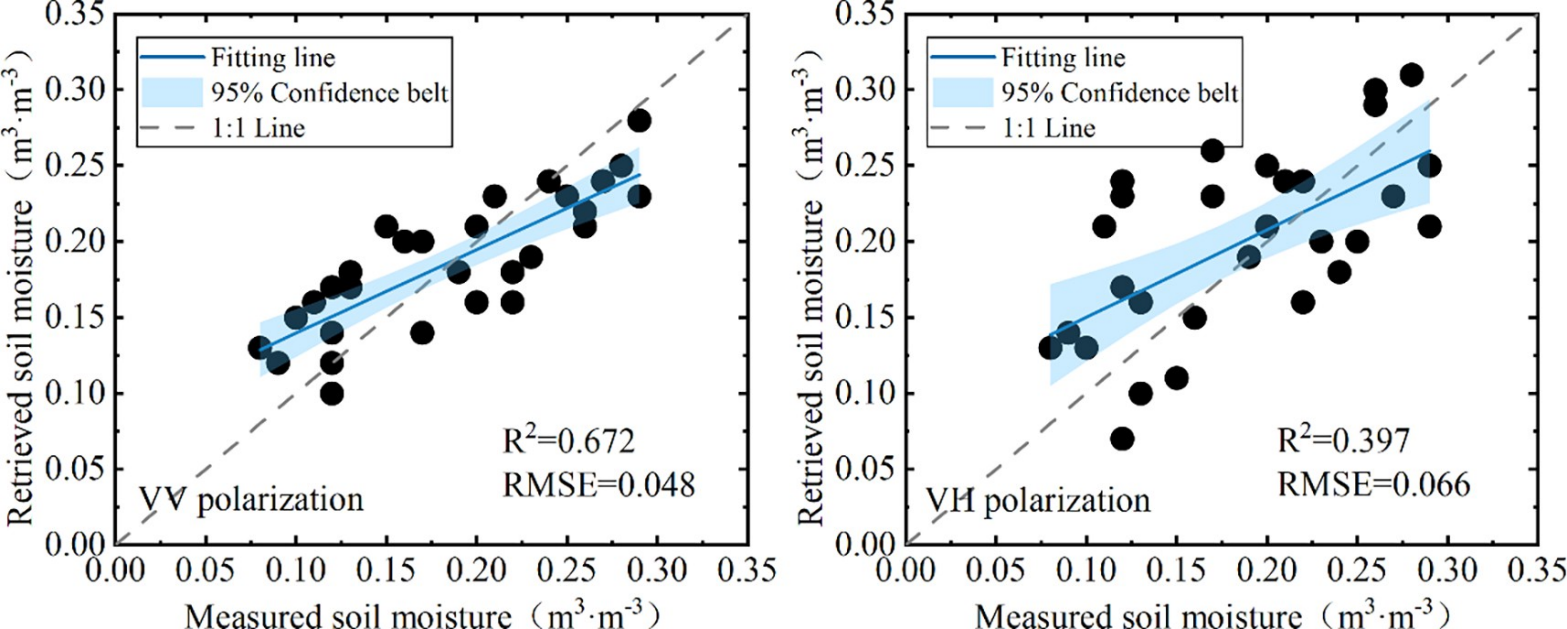
Comparison of the predicted and measured soil moisture under different SAR polarization modes (a. VV polarization; b. VH polarization).

Fig 11 shows the performance of the LUT method in soil moisture retrieval at different SAR polarization modes. It can be seen that the soil moisture retrieval accuracy by the LUT method is significantly higher than that of the direct retrieval method, which indicates that the influence of surface roughness and other parameters on the backscattering coefficient of soil layer cannot be ignored. Through iterative optimization, the LUT method accurately identified soil moisture values corresponding to specific backscattering coefficients, surface roughness parameters and SAR incident angle from large amounts of data simulated by the Oh model, and provided more accurate results in soil moisture retrieval. It is also noticed that compared with the VH polarization method, the VV polarization method is more sensitive to changes in soil moisture and has higher accuracy and applicability in soil moisture SAR retrieval. The validation points are relatively uniformly distributed near the 1:1 line at the VV polarization mode, the R^2^ between the retrieved soil moisture and the measured value was 0.672, and the RMSE was 0.048 m^3^·m^-3^, which presents a high accuracy. For VH polarization mode, the distribution of the validation points was more scattered, the R^2^ between the retrieved soil moisture and the measured value was 0.397, and the RMSE was 0.066 m^3^·m^-3^, which was significantly lower in accuracy than that of the VV polarization mode. In addition, by comparing the fitted line with the 1:1 line, it can be found that there is a small overestimation of the modeled retrieval value compared with the measured value when the soil moisture is low, while the modeled retrieval value is slightly lower than the measured value when the soil moisture is high, which is in agreement with the conclusions of the studies by Mardan [50] and Libin [51].

## Conclusion

Vegetation is the most significant factors affecting the SAR backscattering coefficient from the land surface, which may lead to unreliable results and low accuracy of soil moisture. Using Sentinel-1 SAR remote sensing data and Sentinel-2 MSI optical remote sensing data, an integrated approach is explored for the retrieval of soil moisture over dense vegetated surfaces. Firstly, various vegetation indices derived from Sentinel-2 MSI imagery were used to establish the VWC estimation model, it was found that FVI, NDVI, NDWI2 and NDGI have good correlation relationships with the VWC of dense vegetation. To integrate the advantages of different vegetation indices, a multiple regression model was established with the above four vegetation indices, the model presents better results in the estimation of VWC than the models that constructed with a single vegetation index, with an R^2^ of 0.709, and an RMSE of 0.30 kg·m^-2^. Then the VWC estimation model was coupled with WCM to correct the contribution of the vegetation layer in the SAR backscattering coefficient, and the backscattering coefficient of soil layer was obtained. After vegetation correction, the average decreases of backscattering coefficients at the VV and VH polarization modes were 3 dB and 4 dB, respectively, and the correlations between soil moisture and SAR backscattering coefficients were significantly improved. The correlation coefficient increased from 0.659 to 0.802 for the VV polarization mode, and from 0.398 to 0.509 for the VH polarization mode. Based on the backscattering coefficient simulation database generated by the Oh model, the LUT method was used to retrieval soil moisture, and the results were compared with those obtained by the direct retrieval method. The R^2^ between the retrieved soil moisture and the measured value was 0.672, and the RMSE was 0.048 m^3^·m^-3^ at VV polarization mode, and for VH polarization mode, The R^2^ and RMSE were 0.397 and 0.066 m^3^·m^-3^, the accuracy is significantly higher than that of the direct retrieval method, which has an R^2^ of 0.529 in VV polarization and 0.245 in VH polarization.

The coupled approach employed in this study integrates a VWC estimation model with the WCM, effectively eliminating the contribution of dense vegetation to SAR backscattering coefficients, a satisfactory soil moisture retrieval accuracy was obtained by using the Oh model and the LUT algorithm. The results in this study provide a preliminary validation of the effectiveness of this approach, and proved the potential to combine Sentinel-1 SAR data with Sentinel-2 MSI data to estimate soil moisture over surfaces with dense vegetation coverage. In the future, more work is needed to further explore the applicability of the approach in other regions and over surfaces covered by other types of vegetation. Further, integrating machine learning algorithms into the soil moisture retrieval research is also a direction worthy of exploration.

## Supporting information

S1 Data (XLSX)
